# GENDER AND HOUSEWORK IN THE POSTRETIREMENT CONTEXT

**DOI:** 10.1093/geroni/igad104.1731

**Published:** 2023-12-21

**Authors:** In Jeong Hwang, Joseph Svec, Jeong Eun Lee

**Affiliations:** Harvard University, Boston, Massachusetts, United States; Saint Joseph’s University, New York City, New York, United States; Iowa State University, Ames, Iowa, United States

## Abstract

In recent years, social scientists have noted an uptick in “unretirement” of older adults as a large number of retirees have reentered the labor force. Not only do older Americans’ labor-force reentry have implications for our economy as a whole, this also raises important questions regarding the social and interpersonal relationships within households. Specifically, women, compared to men, might be disproportionately subject to work-family conflicts, due to the gendered expectation that women take primary responsibility for household labor. In this study, we examine 758 American couples aged 65 and above from the 2005-2019 Health and Retirement Study (HRS) core survey and the supplemental Consumption and Activities Mail Survey. We examine whether and to what extent couple’s retirement and re-entry into the labor force correspond with changes in the distribution of household labor. Specifically, we expect that while both men and women shift their household labor contributions in accordance to changes in their formal work statuses, changes in men’s contributions are attenuated relative to women’s. The results show that while both male and female retirees spend more time housework when their spouses work, women tend to perform more housework when they re-enter the labor force whereas men’s contributions decline significantly upon their re-entry. These patterns are increasingly evident from the analysis focused on routine tasks, typically done by women. These results highlight how gender ideological scripts can frame relational contributions and expectations as well as their persistence despite shifts in economic contributions to the household.

